# Limitations in cataract surgical services for children in Ethiopia: a nationwide survey of pediatric cataract surgeons

**DOI:** 10.1186/s12886-021-02190-0

**Published:** 2021-12-19

**Authors:** Mulusew Asferaw, Kumale Tolesa, Sadik Taju Sherief, Bezawit Tadegagne, Mandefro Sintayehu, Addisu Worku, Teshager Wondale, Emebet Girma, Zelalem Gizachew, Clare Gilbert, Geoffrey Woodruff

**Affiliations:** 1 WGGA Eye Center, Pediatric Ophthalmology and Strabismus Unit, Addis Ababa, Ethiopia; 2 Department of Ophthalmology, Jimma University Hospital, Jimma, Ethiopia; 3 Department of Ophthalmology, Menelik II Hospital, Addis Ababa University, Addis Ababa, Ethiopia; 4 Department of Ophthalmology, St Paul’s Hospital Millenium Medical College, Addis Ababa, Ethiopia; 5 Department of Ophthalmology, Bisidimo Hospital, Haramaya University, Harer, Ethiopia; 6 Department of Ophthalmology, Gondar University Hospital, University of Gondar, Gondar, Ethiopia; 7 Department of Ophthalmology, Hawassa Referral Hospital, Hawassa University, Hawassa, Ethiopia; 8 Department of Clinical Research, International Centre for Eye Health, London School of Hygiene & Tropical Medicine, London, UK; 9 Department of Ophthalmology, Leicester Royal Infirmary, Leicester, UK

**Keywords:** Pediatric cataract surgery, Child eye health tertiary facility, Pediatric ophthalmologists, Ethiopia

## Abstract

**Background:**

Bilateral cataract is a significant cause of blindness in children in Ethiopia. This study aimed to identify the resources available for cataract surgery in children, and to assess current surgical practices, surgical output and factors affecting the outcome of surgery in Ethiopia.

**Methods:**

A Google Forms mobile phone questionnaire was emailed to nine ophthalmologists known to perform cataract surgery in young children (0–5 years).

**Results:**

All nine responded. All but one had received either 12- or 3–5-month’s training in pediatric ophthalmology with hands-on surgical training. The other surgeon had received informal training from an experienced colleague and visiting ophthalmologists. The surgeons were based in seven health facilities: five in the capital (Addis Ababa) and eight in six public referral hospitals and one private center.

Over 12 months (2017–2018) 508 children (592 eyes) aged 0–18 years (most < 15 years) were operated by these surgeons. 84 (17%) had bilateral cataract, and 424 (83%) had unilateral cataract mainly following trauma. A mean of 66 (range 18–145) eyes were operated per surgeon. Seventy-one additional children aged > 5 years were operated by other surgeons. There were substantially fewer surgeons per million population (nine for 115 million population) than recommended by the World Health Organization and they were unevenly distributed across the country.

Methylcellulose and rigid intraocular lenses were generally available but less than 50% of facilities had a sharp vitrectomy cutter and cohesive viscoelastic. Mean travel time outside Addis Ababa to a facility offering pediatric cataract surgery was 10 h.

**Conclusion:**

Despite the high number of cases per surgeon, the output for bilateral cataracts was far lower than required. More well-equipped pediatric ophthalmology teams are urgently required, with deployment to under-served areas.

## Background

The prevalence of blindness in children aged 0–15 years is higher in the sub-Saharan African (SSA) Global Burden of Diseases super-region (0.71/1000 children) than in other regions, due to greater exposure to risk factors and lower access to public health interventions and eye care services [1]. In 2020, SSA was the region with the highest number of blind and severely visually impaired and blind children (350,000, excluding refractive error), with approximately 300 affected children per 10 million population [1]. The proportion of blindness in SSA which is avoidable with today’s knowledge is also high, as many of the conditions are amenable to primary preventive measures, such as measles immunization and vitamin A supplementation, and tertiary prevention, such as surgery for congenital and developmental cataract. However, corneal blindness from measles and vitamin A deficiency is declining, largely as a result of public health interventions, and in many countries cataract is now the principal cause of avoidable blindness, estimated to account for 17% of blindness in SSA in 2020 [1]. Similar findings have been reported from Ethiopia, where bilateral cataract was responsible for 33% of severe visual impairment and blindness in a key informant study [2], and 10% in two studies of children attending special education [3, 4].

To address blinding eye diseases in children in low and middle income countries, in 2002 the World Health Organization (WHO) recommended that one tertiary eye care facility for children be established for every 10 million population [5]. Recommendations were also made for minimal staffing requirements, including an ophthalmologist trained in pediatric ophthalmology, and the minimal essential equipment, which included an operating microscope with co-axial illumination and a vitrectomy machine for cataract surgery. Ethiopia currently has a population of 115 million, which indicates that there should be 11 or 12 of these facilities across the country.

In high income countries good visual acuity outcomes following bilateral cataract surgery can be achieved, with at least 50% having an acuity of 6/18 or better after surgery [6, 7]. Good results have also been described from resource poor settings, with more than 80% of children in a study from Nepal having a corrected visual acuity of 6/12 or better [8]. However, outcomes in Africa, where children typically present very late for surgery, vary widely. In two Tanzanian studies, 54 and 62% of children achieved an acuity of 6/18 in the better eye [9, 10] but only 30% of children in Zambia and 25% in South Africa achieved this level of acuity [11, 12]. There are only two studies of the outcome of surgery in Ethiopia. In a recent retrospective study in Gondar, 58% of eyes in children with bilateral cataract achieved 6/18 or better, or fix and follow [13]. In an earlier study in Southern Ethiopia, only 11% of children achieved an acuity of 6/18 or better [14].

In view of the ongoing problem of cataract blindness in children and the variable outcomes in Ethiopia, we initiated the CHICOE project (Children’s Improvement in Cataract Outcome in Ethiopia). This first publication is authored by all nine ophthalmologists undertaking cataract surgery in young children (age 0–5 years) in Ethiopia, and provides a national picture of cataract surgery in children in terms of the training the ophthalmologists had received, the resources available, the volume of surgery and surgical techniques, and ophthalmologists’ views on the main reasons for poor outcomes and delay in surgery.

## Methods

In August 2018, the corresponding author, a pediatric ophthalmologist, emailed a two-part mobile phone survey questionnaire in Google Forms to all nine surgeons known to undertake cataract surgery in young children (aged 0–5 years) in Ethiopia. Part I consisted of 17 questions about the institution where the surgeon operates and their training in pediatric ophthalmology. Part II was a 20-question survey of surgical practices for cataract surgery on children aged 0–18 years, with some questions on surgical techniques on children aged 0–5 years. Questions included the number of eyes operated by each surgeon over the previous 12 months, the youngest age at which they implant an intraocular lens, and the availability of equipment and consumables. Surgeons were also invited to comment on factors they judged to have limited good outcomes of the surgery over the previous year, and the relative importance of different causes of delay in surgery.

A third questionnaire consisting of 14 supplementary questions was developed based on the responses to the first two questionnaires. Each surgeon was asked if they were aware of any other surgeons or institutions in Ethiopia undertaking cataract surgery in children. They were asked what proportion of children age 0–18 years with cataract were operated by themselves, other participants in this study, or by other ophthalmologists.

## Results

All nine surgeons believed to undertake cataract surgery in children aged 0–5 years responded to the questionnaire, and none were aware of any others operating on this age group. Surgeons in four of the centres reported that other surgeons performed surgery on older children (aged > 5 to 18 years).

### Centers undertaking cataract surgery in young children

**Fig. 1:**
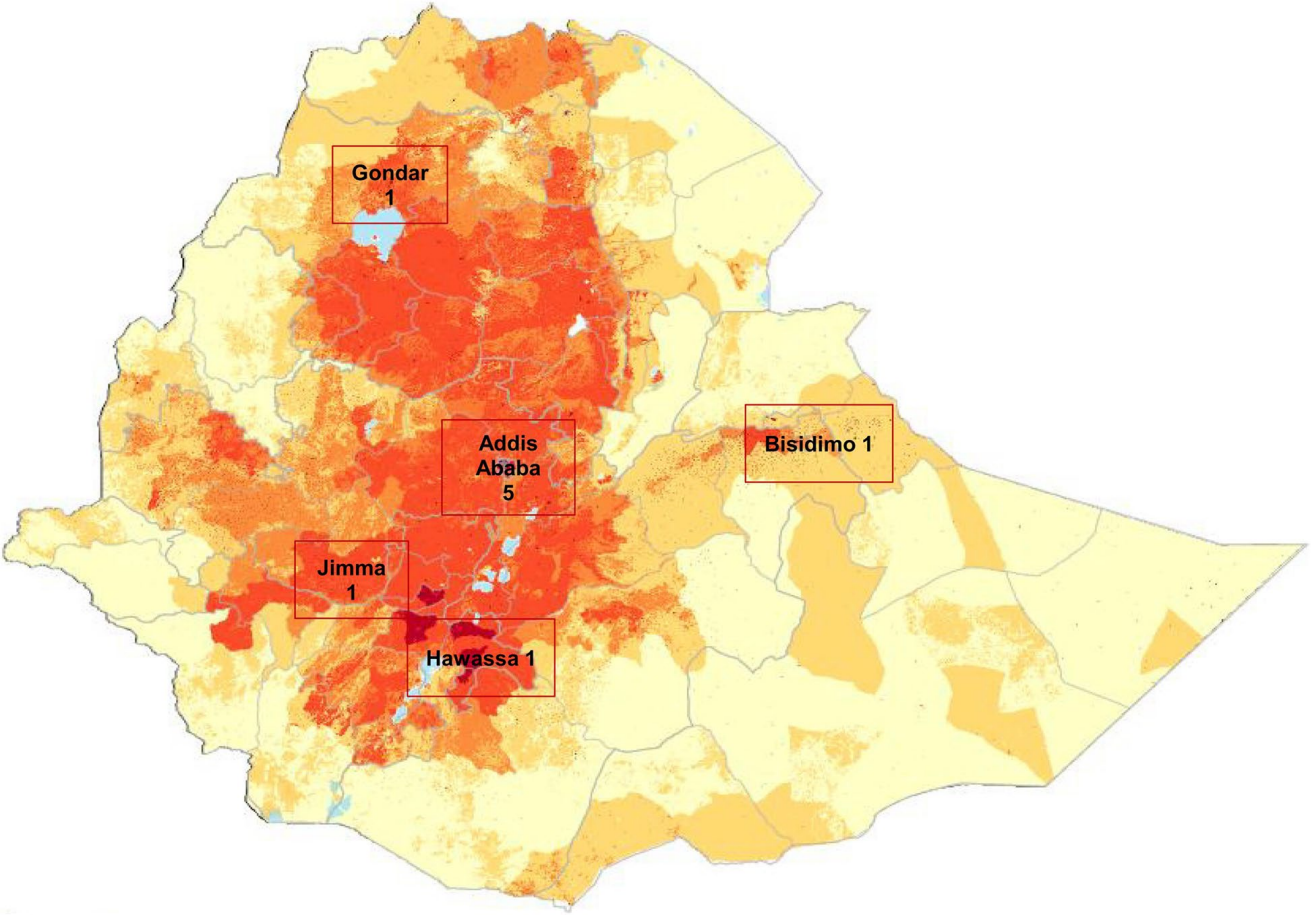
Map of population density of Ethiopia showing the location of the seven facilities where the study ophthalmologists work (https://commons.wikimedia.org/wiki/File:Ethiopia_Population_density_1km_source_AfriPop_10v4.pdf). [Adapted from©Amibretonhttps://en.wikipedia.org/wiki/Creative_Commons/https://commons.wikimedia.org/wiki/File:Ethiopia_Population_density_1km_source_Af_riPop_10v4.pdf/CreativeCommons/CommonsAttribution-ShareAlike3.00Unported]

The surgeons were based in seven facilities: five government university teaching hospitals (two in Addis Ababa and three in peripheral locations (Gondar, Jimma and Hawassa), one government non-teaching hospital (Bisidimo) and one private facility (Addis Ababa) (Table 1, Fig. 1). Five worked in two government hospitals and a private facility in the capital city, Addis Ababa. Among the nine regional states and two city administrations during study period in Ethiopia, only three states and one city have a center which undertakes cataract surgery in young children. For parents of children with cataract who live outside Addis, the mean travel time to another facility offering cataract surgery was 10.2 h.

**Table 1:** Pediatric cataract surgical centers in Ethiopia

Name of center	Location (City/Region)	Type of hospital	Children aged 0–18 years having cataract surgery in previous 12 months	Number of children operated by non-participating surgeons	Number of participating surgeons undertaking cataract surgery on children 0–5 years	Travel time from base hospital to nearest institution offering pediatric cataract surgery (hours)
Menelik II Hospital	Addis Ababa	Government University Hospital	159	42	3	1
St Paul’s Millennium Medical College Hospital	Addis Ababa	Government University Hospital	40	0	2*	1
WGGA Eye Centre	Addis Ababa	Private Centre	6	0	1*	1
Bisidimo Hospital	Bisidimo, Oromia	Government Hospital (non-teaching)	25	10	1	12
Gondar University Hospital	Gondar, Amhara	Government University Hospital	142	17	2*	14
Jimma University Hospital	Jimma, Oromia	Government University Hospital	127	2	1	10
Hawassa Referral Hospital	Hawassa, SNNPR	Government University hospital	80	0	1	5

*One study surgeon operated at two other centers in addition to their base center

*SNNPR* Southern Nations, Nationalities, and Peoples’ Region

### Surgical output

A total of 508 children aged 0–18 years were operated for cataract by the participating surgeons between September 2017 and August 2018. The surgeons commented that they had extracted data from surgical logbooks, and majority were aged less than 15 years of age. 84 (17%) children had bilateral, and 424 (83%) had unilateral cataract, mainly following trauma. A total of 592 eyes were operated, giving a mean of 66 (range 18–145 standard deviation (SD) ±47) eyes per surgeon. At four institutions a further 71 older children aged > 5–18 years were operated on by other surgeons, including 12 operations by visiting pediatric ophthalmologists from high income countries (Table 1).

### Availability of equipment and consumables

**Fig. 2:**
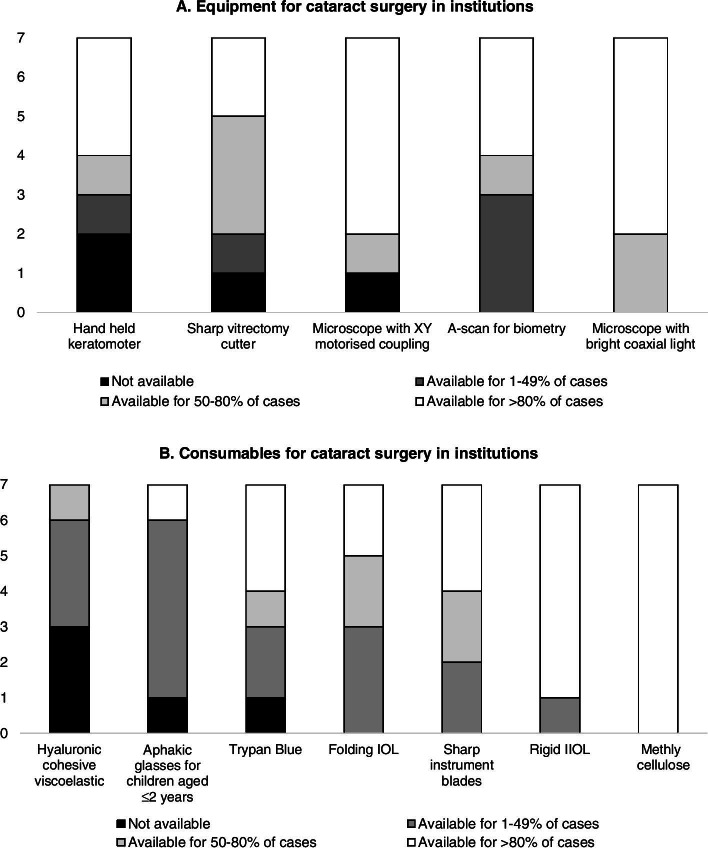
Availability of Equipment (**A**) and Consumables (**B**) in the institutions

There was no clear association between the availability of consumables and equipment and location i.e., in or outside Addis Ababa. The availability of a sharp vitrectomy cutter was as follows: for > 80% of cases in the private centre in the capital and in one of the peripheral centres; for 50–80% of cases in the remaining peripheral hospitals; and in the capital for 1–49% of cases in one centre, and never available in the other centre (Fig. 2A).

Surgeons in all three facilities in Addis and in two peripheral facilities always had access to a microscope with X-Y shift capable of providing a bright red reflex. However, a surgeon in a government hospital in Addis reported seeing a red reflex in only 50–80% of cases, and another surgeon in a peripheral facility reported that the microscope was only available for 50–80% of bilateral cases (Fig. 2B). A peripheral centre had a microscope that consistently provided a bright red reflex but lacked X-Y shift.

In all facilities methylcellulose was available for > 80% of bilateral cases operated over the previous year (Fig. 2A). The availability of hyaluronic cohesive viscoelastic was as follows: never available in three facilities (2 in the capital, 1 in peripheral); available for 1–49% of cases in three facilities (2 in peripheral, 1 in the capital); and for 50–80% in one peripheral center.

Rigid intraocular lenses (IOL) were available for > 80% of surgeries in six of the seven facilities and foldable IOLs were available for > 80% of cases in only two centers. The availability of other equipment and consumables varied, with handheld keratometry, Trypan blue and aphakic glasses never available in at least one facility (Fig. 2A).

### Training and surgical techniques

Six of the nine surgeons had completed a one-year pediatric ophthalmology fellowship, but the opportunities for hands on surgery during this training varied (Table 2). Two surgeons were enrolled in a fellowship training program at the time of the survey but had not yet completed this. Another surgeon had not had formal fellowship training but was being trained and mentored by one of the other participating surgeons and visiting ophthalmologists from high income countries.

**Table 2:** Training and experience of nine Ethiopian participating pediatric cataract surgeons

Surgeon / year qualified	Duration (months) and location of training in pediatric ophthalmology				Number of Pediatric cataract surgeries				Surgical techniques	
	Fellowship or post residency training (months)	Hands-on surgical training (months)	Country where trained	Months since completion of training	Under supervision	Total career	Operations in previous 12 months		Youngest age at IOL implantation (years)	Age after which PPC not required (years)
						Eyes	Eyes	Children with bilateral cataract		
A, 2011	8	5	Nepal	Not complete	10	100	18	3	2	8
B, 2016	12	11	Tanzania	7.5	40	290	137	18	1.2	8
C, 2010	12	12	Tanzania	52.4	200	701	100	20	1.5	10
D, 2018	5	3	South Korea and Nepal	Not complete	37	192	145	20	0.3	10
E, 2011	12	5	USA and Nepal	7.5	40	120	32	2	2	5
F, 2011	13	13	Canada	24.5	30	330	27	1	1	5
G, 2010	12	5	Nepal, Israel and USA	7.3	600	750	74	13	0.6	5
H, 2011	3	3	Mentoring at place of work	Not Complete	10	27	24	2	2	8
I, 2010	12	12	Tanzania and Nepal	46.5	150	950	35	5	0.3	8
Average	9.9	7.7		24.3	124.1	384.4	65.8	9.3	1.2	7.4

*IOL* Intraocular lens, *PPC* Primary posterior capsulotomy

A mean of 384 (range 27–950) eyes had been operated for cataract in children aged 0–18 years by the nine surgeons throughout their careers. The youngest average age of children at which surgeons had implanted an IOL was 14 months (range 4–24 ± 8.0) and the mean age beyond which surgeons judged primary posterior capsulotomy not to be required was 7.4 (range 5–10 ± 1.9) years (Table 2).

**Fig. 3:**
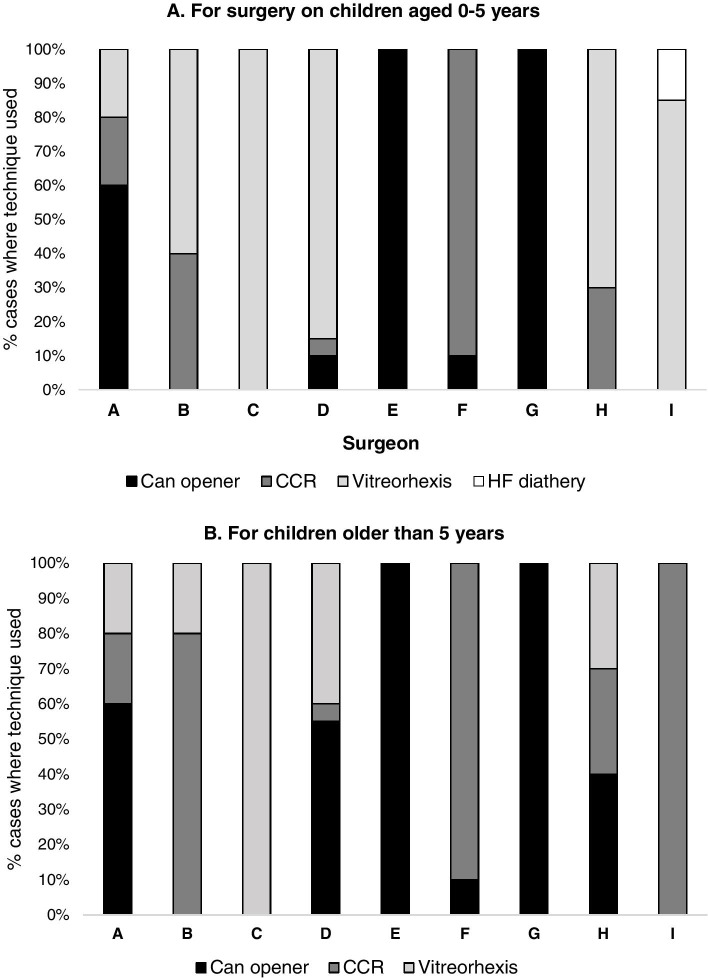
Techniques used for managing the anterior capsule **A**. In children aged 0–5 years **B**. In children older than 5 years by nine surgeons

There was a wide range in techniques for anterior capsulotomy in children aged 0–5 years: two surgeons used a can opener technique in all cases, one used vitreorhexis in all cases and one used high frequency diathermy in 15% of cases (Fig. 3A). Four surgeons never or almost never used manual curvilinear continuous capsularrhexis (CCCR) even in older children (Fig. 3B). These four surgeons included one who had completed training more than 4 years earlier, and two who had completed a one-year fellowship but with only three or 5 months hands-on surgical training.

### Factors affecting outcome of surgery for bilateral cataract over the 12 months

**Fig. 4:**
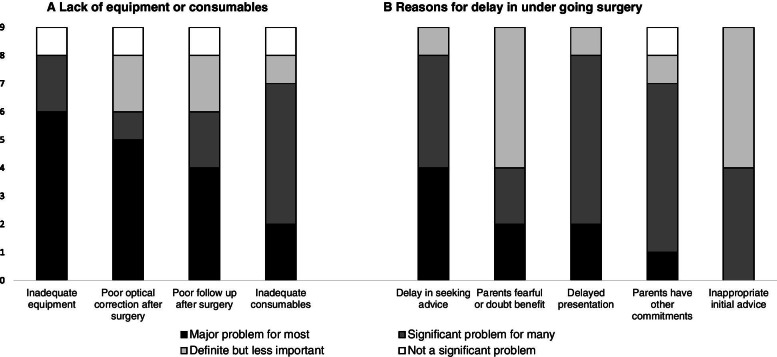
Factors affecting the outcome of surgery **A**. Lack of equipment **B**. Reason for delayed surgery

Eight of the nine surgeons stated that inadequate equipment or consumables had been a significant or major problem limiting the outcome in most or many children (Fig. 4A and B). Five reported post-operative optical correction as a major problem in most cases and two volunteered that a major limiting factor was the lack of regular access to paediatric anaesthesia.

All nine surgeons judged that delay in surgery was the most important factor which limited the outcome of surgery for children with bilateral cataract. They reported that delays occurred at every step, from delayed recognition of a problem by parents, delay in seeking advice, inappropriate initial advice and other parental commitments.

## Discussion

This study has shown that useful information can be gained by a Google Forms survey of surgeons in a resource poor environment.

We identified nine paediatric cataract surgeons working in seven facilities providing cataract surgery for children, three in the capital city Addis Ababa, and four outside the capital. These centres serve a total population of 115 million i.e., one for every 16.4 million people, which is lower than the WHO recommendation of one Child Eye Health Tertiary Facility (CEHTF) with well trained staff and adequate resources for every 10 million population by the year 2020 [5]. The services are also very poorly distributed. The two government facilities in the capital city serve a population of approximately 8 million as well as some patients from outside the capital region who travel to Addis for surgery. In contrast, the four government centres outside Addis, each with one surgeon, nominally serve the rest of the population (an average of one per 26.8 million people). The northern part of the country is particularly under-served. Addressing this maldistribution of resources will be challenging. However, the essential packages for eye care being developed by WHO as an integral component of Universal Health Coverage will include congenital cataract, which may improve human resource planning for eye care in children [15]. This development provides an opportunity for advocacy with the Ministries of Health in Ethiopia for more resources for child eye care services. Additional services outside the capital city would reduce travel and costs for parents, and promote earlier access, which is important as surgeons in this study reported that delay in presentation was the major cause of poor outcomes.

All but one of the surgeons had undergone formal training in cataract surgery for children, and the total number of cataract operations performed by each surgeon indicates extensive surgical experience. Recently there has been substantial support of fellowship training in pediatric ophthalmology in Ethiopia by two international non-government organizations, Orbis International and the Himalayan Cataract Project, with at least one established pediatric cataract surgeon now contributing to the training of newly appointed surgeons. This reflects what was reported in a baseline and follow up study of tertiary level child eye care facilities in SSA, which demonstrated that staffing and infrastructure had improved significantly between the two studies [16, 17].

The method used for anterior capsulotomy gives an indication of the sophistication of the techniques surgeons were exposed to during training. In this study four surgeons never or almost never used CCCR even in older children and two performed can-opener capsulotomy in almost all cases. This is likely explained by the fact that most of the surgeons had been taught anterior capsulotomy using a vitrector rather than CCCR. In addition, because small incision cataract surgery is used for adults in Ethiopia which does not require CCCR, unlike phacoemulsification, they would not have gained skills in CCCR by operating on adults. The lack of capsulorrhexis forceps suitable for use through a small corneal incision may also have contributed to the relatively infrequent use of CCCR. There is a clear a need for further training with provision of relevant equipment, which all the surgeons would welcome. The recently proposed pediatric ophthalmology academic partnership between Canada and Ethiopia could help to address these gaps [18].

Compared to national studies from high income countries, the number of cases operated per surgeon in our study was very high. For example, in a survey of US paediatric cataract surgeons in 2003, 86% operated on less than 21 eyes a year [19] compared with our study where the mean number of eyes operated per surgeon was 66. This reflects the low number of pediatric ophthalmologists, with only one surgeon for every 12.8 million population. The volume of cataract surgery by the private facility in Addis was very small although this surgeon contributes to teaching and providing services in two government facilities. The cataract surgical output is not adequate for the need, as unoperated cataract is a significant cause of blindness in children in many low income countries, including Ethiopia, as identified in a population based study and a study of children attending schools for the blind [2, 4]. A study in Malawi reported that poverty and long distances to eye care facilities were the main reasons why parents did not accept surgery for their child, even when offered free of cost, and the same is likely to apply in Ethiopia [20].

Our surgeons reported that two thirds of the children they operated on had unilateral cataract, mostly following trauma, which is a higher than in studies in high income countries (25–30%) [7]. Other studies in Africa have also shown that serious ocular injury in children is very common, and is often caused by playing with sticks [21]. Preventive measures, such as health education in schools, or ‘toy for stick’ exchange programs could reduce this morbidity.

Surgeons often cited “other parental commitments” as contributing to delayed surgery, which is likely to be exacerbated by uneven distribution of services and the long travel distances required to access them. This is consistent with other studies of inequalities in access to health care [22] and suggests that many children with visually significant cataract in Ethiopia never present for surgery.

Our study has limitations as it was questionnaire based rather than observational and relied on data collected retrospectively and recall of surgeons’ experiences. Moreover, in a rapidly changing situation, this survey provides only a snapshot for the year 2017/2018. However, in the absence of other information about pediatric cataract surgical practice in Ethiopia, our study provides the first information on the limitations of these services.

## Conclusions

The number of CEHTFs is lower than the WHO recommendation, and the services that are available are poorly distributed across the country. It is, therefore, not surprising that late presentation is an important cause of poor outcomes. Many centers lack the equipment and consumables considered essential for high quality cataract surgery in young children.

There is a need to increase the number and distribution of pediatric ophthalmologists and ensure they are adequately trained and equipped to provide high quality surgery. A minimum of five more CEHTFs with the full complement of competent allied eye health professionals are required to meet WHO recommendations. Advocacy with the Ministry of Health to address these challenges is very important, working in partnership with NGOs.

We propose that this survey is followed by a retrospective review of the visual acuity outcome of bilateral cataract surgery in Ethiopian children to provide baseline data, followed by a prospective national study following the provision of additional resources.

## Data Availability

The datasets generated during and analyzed during the current study are not publicly available due to presence of personal identification of participants, but are available from the corresponding author on reasonable request.

## Abbreviations

CCCR: Curvilinear continuous capsular rhexis
CEHTF: Child eye health tertiary facility
CHICOE: Children’s Improvement in Cataract Outcome in Ethiopia
IOL: Intraocular lens
NGO: Non-governmental organization
PPC: Primary posterior capsulotomy
SD: Standard deviation
SSA: Sub-Saharan Africa
WHO: World Health Organization
